# Oxidative Stress and Neurodegenerative Disorders

**DOI:** 10.3390/ijms141224438

**Published:** 2013-12-16

**Authors:** Jie Li, Wuliji O, Wei Li, Zhi-Gang Jiang, Hossein A. Ghanbari

**Affiliations:** 1 Department of Geratology, First Hospital of Jilin University, Changchun, Jilin 130021, China; E-Mail: yabianwanghai@163.com; 2 College of Pharmacology, Inner Mongolia University for the Nationalities, Tongliao, Inner Mongolia 028000, China; E-Mail: wuliji@126.com; 3 Cancer Center, First Hospital of Jilin University, Changchun, Jilin 130021, China; E-Mail: drweili@yahoo.com; 4 Panacea Pharmaceuticals, Inc., Gaithersburg, MD 20877, USA; E-Mail: hag@panaceapharma.com

**Keywords:** aging, oxidative stress, neurotoxicants, signal transduction, neurodegenerative diseases, chemobrain, peripheral neuropathy, pharmaceutical

## Abstract

Living cells continually generate reactive oxygen species (ROS) through the respiratory chain during energetic metabolism. ROS at low or moderate concentration can play important physiological roles. However, an excessive amount of ROS under oxidative stress would be extremely deleterious. The central nervous system (CNS) is particularly vulnerable to oxidative stress due to its high oxygen consumption, weakly antioxidative systems and the terminal-differentiation characteristic of neurons. Thus, oxidative stress elicits various neurodegenerative diseases. In addition, chemotherapy could result in severe side effects on the CNS and peripheral nervous system (PNS) of cancer patients, and a growing body of evidence demonstrates the involvement of ROS in drug-induced neurotoxicities as well. Therefore, development of antioxidants as neuroprotective drugs is a potentially beneficial strategy for clinical therapy. In this review, we summarize the source, balance maintenance and physiologic functions of ROS, oxidative stress and its toxic mechanisms underlying a number of neurodegenerative diseases, and the possible involvement of ROS in chemotherapy-induced toxicity to the CNS and PNS. We ultimately assess the value for antioxidants as neuroprotective drugs and provide our comments on the unmet needs.

## 1. Introduction

Oxygen is required for energy metabolism for the survival and normal functions of most eukaryotic organisms. Along the respiratory chain, oxygen is also partially reduced, at low ratio, into superoxide, a basic free radical that can be converted eventually into other forms of reactive oxygen species (ROS). Cell metabolism could generate other free radicals from nitrogen, classified into the family of reactive nitrogen species (RNS). ROS and RNS at physiological concentrations have recently been demonstrated to mediate a variety of normal functions, such as regulation of signal transduction, induction of mitogenic response, and involvement in defense against infectious agents, *etc.*

ROS are balanced with antioxidant systems to keep their level constant in living organisms. These antioxidant systems are both enzymatic and non-enzymatic. Breaking the balance by over production of ROS and/or reduction of antioxidants can be deleterious, and is termed oxidative stress. Under these conditions, excessive free radicals could freely pass through the plasma membrane, damaging the cell membrane via lipid peroxidation, modifying signal and structural proteins to lead to misfolding and aggregation, and oxidizing RNA/DNA to interrupt transcription and result in gene mutation.

In its lifespan, a living organism could be exposed to a number of oxidative damage-causing exogenous factors, such as irradiation by UV light, X-rays, gamma-rays, heavy metals, and atmospheric pollutants. As we age, the oxidative and/or nitrosative damages elicit a number of late-onset diseases after ROS/RNS accumulate to certain levels. The ROS/RNS-mediated late-onset diseases can occur in any system of the body and in many presentations, such as cancer, arteriosclerosis, arthritis and neurodegenerative diseases. Amongst the different organs in the body, the brain is particularly vulnerable to oxidative stress due to its high oxygen utilization, weaker antioxidant enzymes, high content of easily oxidized polyunsaturated fatty acids, and the terminal-differentiation characteristic of neurons.

This review focuses on the role of oxidative stress on neurodegenerative diseases. To aid the understanding of toxic targets in neurodegenerative diseases, this review begins with the essential characteristics of ROS, including its generation, regulation and physiological functions. Then, the mechanisms for ROS underlying neurodegeneration are highlighted with a focus on the causal relationship between ROS and protein misfolding and aggregation (which can serve as a key to distinguish one neurodegenerative disease from another). In addition, the role of ROS in artificial event-induced neuronal disorders, such as chemotherapy-induced cognitive impairment (colloquially known as chemobrain) and chemotherapy-induced peripheral neuropathy (CIPN) is assessed. The review further comments on drug developmental strategies for the therapy of neurodegenerative diseases, as well as prevention of anticancer drug-induced neuronal disorders. The review closes with an evaluation of methodology that has been applied to measure oxidative/nitrosative stress.

## 2. Reactive Oxygen Species (ROS)

ROS include free radicals, such as the superoxide anion radical (O_2_^•−^), hydroxyl radicals (^•^OH), and the nonradical hydrogen peroxide (H_2_O_2_). Since reactive nitrogen intermediates (RNI) are involved in the regulation of apoptotic or necrotic cell death, they are also recognized as important radicals. These include different forms of nitric oxide (NO^•^), nitroxyl anion (NO^−^) and nitrosonium cation (NO^+^), and peroxynitrite (ONOO^−^).

### 2.1. Productions of ROS and RNI

**Figure 1.**
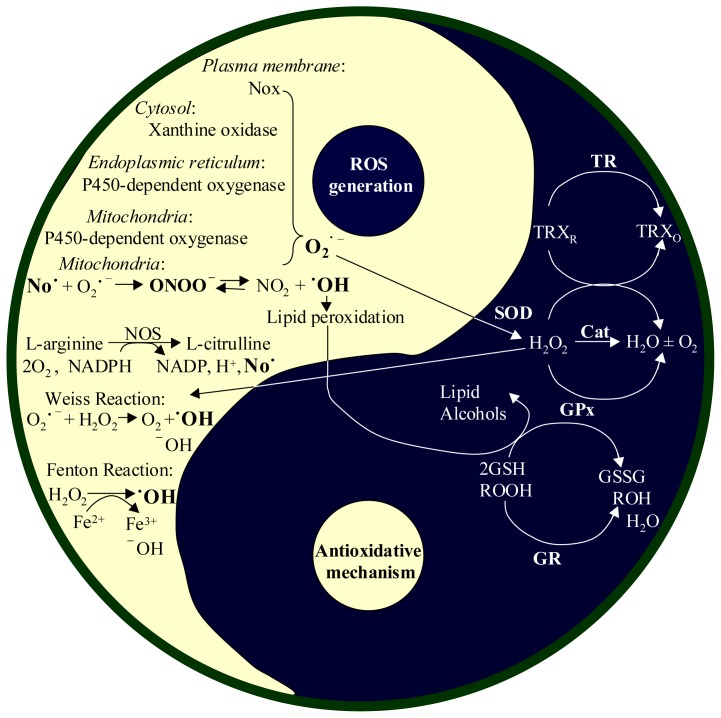
ROS generations and antioxidant systems in cells. Reactive oxygen species (ROS) can be generated from various sites in a cell. The cell protects its own damage from excessive ROS by suppression of ROS levels by two redox buffers (glutathione (GSH) and thioredoxin (TRX)) and antioxidant enzymes, including superoxide dismutase (SOD), catalase (CAT), thioredoxin reductase (TR), and glutathione peroxidase (GPx). In the cell, superoxide dismutase (SOD) first converts O_2_^•−^ into H_2_O_2_, and then catalase (Cat) enzymatically converts H_2_O_2_ is into H_2_O and O_2_. In the GSH buffer system, GPx converts H_2_O_2_ into H_2_O and O_2_ when it converts GSH into its oxidized disulfide form (GSSG). GSSH is then reduced by glutathione reductase (GR) to regenerate GSH for reuse. In the TRX buffering system, the TRX in reduced status (TRX_R_) is oxidized into oxidized thioredox (TRX_O_) during the degradation of H_2_O_2_ and then reduced by TR. Besides ROS, No^•^ is also enzymatically generated by nitric oxide synthases (NOS), and is further reacted with O_2_^•−^ to produce ONOO^−^.

ROS can be generated in the mitochondria, endoplasmic reticulum (ER), plasma membrane and cytoplasm (Figure 1). Cells constantly generate ROS in mitochondria during aerobic metabolism. Under normal condition, 1%–2% of electrons leak from the mitochondrial electron transport chain and form O_2_^•−^ by cycling the ubiquinol in the inner mitochondrial membrane. NADH-ubiquinone oxidoreductase (Complex I) and ubiquinol-cytochrome c oxidoreductase (Complex III) are the two sites for O_2_^•−^ production [1]. O_2_^•−^ is enzymatically produced by cytochrome P450-dependent oxygenases in the ER of the liver, lung and small intestine [2,3] and NADPH oxidase (Nox) located on the cell membrane of phagocytes as well [4,5]. In the cytosol, xanthine oxidase (XO) provides another enzymatic source to produce both O_2_^•−^ and H_2_O_2_[6]. In addition, O_2_^•−^ is produced non-enzymatically by transferring a single electron to oxygen by reduced coenzymes, prosthetic groups (e.g., flavins or iron sulfur clusters) or previously reduced xenobiotics [1].

O_2_^•−^ is the precursor of most ROS and a mediator in oxidative chain reactions. It is spontaneously converted or catalyzed by superoxide dismutases (SOD) into H_2_O_2_, which is then partially reduced to ^•^OH in the presence of Fe^2+^ by the Fenton reaction [7]. ^•^OH can also be generated via the metal-catalyzed Haber-Weiss reaction.

NO^•^ is generated from l-arginine by a family of enzymes called nitric oxide synthases (NOS), including neuronal NOS (nNOS), endothelial NOS (eNOS) and inducible NOS (iNOS), which are all located in the cytosol. In contrast to these, mitochondrial NOS (mtNOS), the α-isoform of nNOS, is located in the mitochondria where co-existence of NO and O_2_^•−^ results in the formation of ONOO^−^. NO^−^ comes from a reaction of NO with Heme-Fe^2+^, and NO^+^ is derived from a reaction of NO with Heme-Fe^3+^[8,9].

### 2.2. Antioxidant Systems in the Body

To limit over-accumulation of ROS in the body, there exists both enzymatic and non-enzymatic systems to maintain ROS balance. Enzymatic antioxidant defenses (Figure 1) include SOD, glutathione peroxidase (GPx), thioredoxin reductase (TR) and catalase (CAT) [10]. SOD converts O_2_^•−^ to O_2_ and H_2_O_2_. GPx further destroys peroxides (H_2_O_2_ and organic peroxide ROOH) to form H_2_O in the presence of the tripeptide glutathione (GSH). Thioredoxin reductase (TR) is also essential for keeping low levels of H_2_O_2_ by converting it into H_2_O and O_2_ as well [11,12]. CAT, another enzyme that could convert H_2_O_2_ to H_2_O and O_2_, is present in the cells of all aerobic bacteria, plants and animals. In addition to the enzymatic defense systems, the human body also uses non-enzymatic antioxidants to limit over-accumulation of ROS. These include, but are not limited to, ascorbic acid (vitamin C), α-tocopherol (vitamin E), glutathione (GSH) and flavonoids [13]. Vitamin C is a potent antioxidant that neutralizes free radicals by donating an electron. Vitamin E is a fat-soluble vitamin whose main antioxidant function is protection against lipid peroxidation, providing a high efficiency antioxidant effect by stopping ROS from forming in membranes undergoing lipid peroxidation. GSH is highly abundant in the cytoplasm, nuclei and mitochondria. GSH reacts with a radical and becomes a thiyl radical itself. The newly-generated thiyl radicals dimerize to form the non-radical product oxidized glutathione (GSSG). GSH in the nucleus maintains the redox state of sulfhydryls of critical proteins for DNA repair and gene expression. Flavonoids constitute the most important single group of polyphenols, acting as antioxidants by terminating free radical chain reactions. Flavonoids stop the oxidation of lipids and other molecules by the rapid donation of hydrogen atoms to radicals, becoming the phenoxy radical intermediates by themselves. The intermediates are relatively stable, and thus do not initiate further radical reaction. There are also many other non-enzymatic antioxidants in the body, such as selenium, carotenoids, lipoic acid, coenzyme Q, melatonin, *etc*, which are detailed in recent reviews [10,13].

### 2.3. Physiological Functions of ROS

Although the deleterious effects of ROS and RNI are well known, a growing body of evidence in recent years has revealed that ROS or RNI at low/moderate concentrations play important roles in normal physiological processes and body defenses, providing beneficial effects. ROS involves a number of cellular signaling pathways in controlling cell survival, migration and proliferation. H_2_O_2_, the end product of O_2_^•−^ degeneration, serves as a signal molecule through oxidative modification of signaling proteins. Akt (also known as protein kinase B or PKB) is a serine/threonine kinase that predominantly mediates the signal pathway of cell survival by direct inhibition of pro-apoptotic regulators such as Bad, and mitotic-related transcription factors such as Myc. Protein phosphatase 2A (PP2), also known as PP2A has been demonstrated to be a key Akt phosphatase, which dephosphorylates Akt on both threonine 308 and serine 473, blocking the PI3K/Akt pathway. H_2_O_2_ can inactivate PP2A at the cysteine site and therefore activate the Akt pathway to facilitate cell survival [14–16]. Mitogen-activated protein kinases (MAPK) are another family of serine/threonine kinases. Among them, the extracellular signal-regulated kinase (ERK) pathway has most commonly been associated with the regulation of cell proliferation. In this pathway, MAPK kinase kinases (MAPKKK) phosphorylate and activate MAPK kinases (MAPKK or MAPK/ERK kinases (MEKs)), which in turn phosphorylate and activate MAPKs (or ERKs) and eventually facilitate cell proliferation. The negative regulation of this pathway is fulfilled by dephosphorylation of MAPKs by phosphatases. ROS activates the ERK signal in several ways. Firstly, the ERK pathway is downstream of EGF and PDGF receptors. Oxygen radicals elicit phosphorylation of these receptors, resulting in activation of the ERK pathway in relation to mitogenic signaling [17–19]. In fact, the activation of the ERK pathway is not limited to receptor levels. ROS can activate Src kinase upstream of RAS, leading to activation of the ERK signal [20]. The ERK pathway is also activated by the direct inhibition of MAPK phosphatases with hydroxyl radicals [21,22]. ROS actively mediates cell differentiation. Myogenic differentiation is an essential process for myogenesis in response to various extracellular stimuli, including ROS.

Apoptosis signal-regulating kinase (ASK) 1, a member of the MAPKKK family, is an intracellular inducer of p38 MAPK-mediated myogenic signaling in cardiac myoblasts. An array of stresses such as oxidative stress can activate ASK1 and initiate the differentiation process of myoblasts [23]. As a signaling molecule, ROS has been shown to mediate the function of angiogenic factors like VEGF or angiopoietin-1 in directing cell migration [24]. In addition to the regulation of cellular signaling pathways as secondary messengers, ROS are also involved in cell defense against infectious agents. Under an inflammatory condition, activated neutrophils and macrophages produce large quantities of superoxide radical and other ROS via the phagocytic isoform of NAD(P)H oxidase, where the concentration of H_2_O_2_ may reach 10–100 μM to facilitate the defense requirement [25].

**Figure 2.**
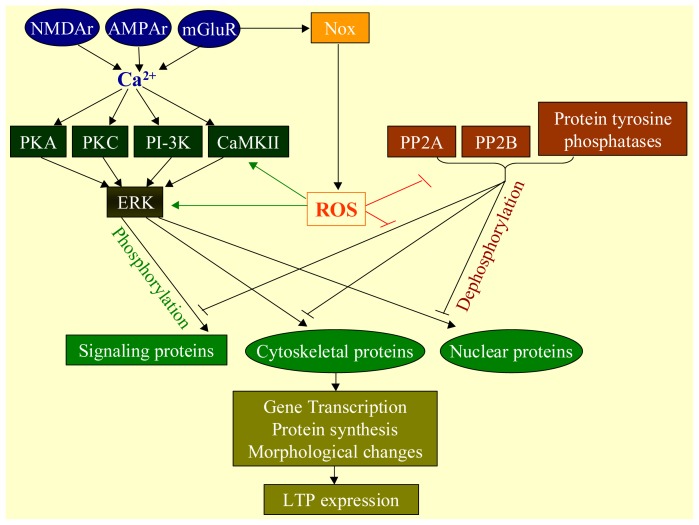
Contribution of ROS to the process of learning and memory. Long-term potentiation (LTP) is considered one of the major cellular mechanisms for learning and memory. It is positively regulated by activation of glutamate receptors, including *N*-methyl-d-aspartate receptor (NMDAr), α-amino-3-hydroxy-5-methyl-4-isoxazolepropionic acid receptor (AMPAr) and metabotropic glutamate receptor (mGluR). The activation of these receptors results in calcium influx, which then activates different kinases in the cascade to facilitate LTP formation. In contrast, LTP is negatively regulated by phosphatases, including protein phosphatase 2A and 2B (PP2A and PP2B). Activation of glutamate receptors result in O_2_^•−^ accumulation, possibly through the conversion of NADPH oxidase (Nox).

Oxidative stress contributes to physiological functions of the CNS, including the process of learning and memory (Figure 2). Long-term potentiation (LTP) is a long-lasting enhancement in signal transmission between two neurons that results from stimulating them synchronously. As memories are thought to be encoded by modification of synaptic strength, LTP is widely considered as one of the major cellular mechanisms that underlie learning and memory.

The pathways to activate LTP are different, depending on the hippocampal regions. For example, LTP in the Schaffer collateral pathway is *N*-methyl-d-aspartate receptor (NMDAr) -dependent, whereas LTP in the mossy fiber pathway is NMDA receptor-independent [26]. In other brain regions, metabotropic glutamate receptor (mGluR)-dependent LTP presents in the cerebellar cortex at parallel fibers to Purkinje cell synapses [27]. The activation of NMDA receptors admits free calcium (Ca^2+^) into the postsynaptic neuron, and buffering a rinse in Ca^2+^ prevents cell membrane leakage [28]. Calcium influx results in the phosphorylation of downstream signaling kinases, such as phosphoinositide 3-kinase (PI-3K) [29], protein kinase A (PKA) [30,31] and protein kinase C (PKC) [32–34] and calcium/calmodulin-dependent protein kinase II (CaMKII) [35–38]. These enzymes in active condition further activate downstream ERK. Upon activation, ERK may phosphorylate a number of cytoplasmic and nuclear molecules that ultimately result in the protein synthesis and morphological changes necessary for formation of LTP [39]. ERK-mediated changes in transcription factor activity may trigger the synthesis of proteins that underlie the maintenance of LTP. One such molecule may be protein kinase Mζ (PKMζ), an atypical isoform of PKC whose synthesis increases following LTP induction [40,41]. PKMζ thus appears important for the persistence of memory and would be expected to be important in the maintenance of long-term memory since PKMζ becomes a requirement for LTP maintenance only during the late phase of LTP [40]. Considerable evidence has also shown that certain forms of LTP induction at excitatory synapses are dependent on activation of mGluRs that are widespread in different brain regions such as the neocortex, hippocampus, striatum and nucleus accumbens. The activation of mGluR can activate a range of intracellular mediators including protein kinase C (PKC) and protein kinase A (PKA), the tyrosine kinase Src and nitric oxide (NO) [42], perhaps in relation to calcium influx [43]. ROS are considered cellular messengers in the formation of LTP. Superoxide accumulates in rodent hippocampal slices after NMDA receptor activation [44] and plays an important role in LTP. Scavenging superoxide in hippocampal slices blocks high-frequency stimulation-induced LTP [45]. Genetic deletion of NADPH oxidase subunits results in deficient LTP [46]. Incubation of hippocampal slices with exogenous superoxide dismutase (SOD) attenuates LTP [47]. ROS can induce LTP by modulating the activity of a number of protein kinases such as PKA, PKC, CaMKII and ERK [48–50]. In addition, ROS can also induce LTP through suppression of the activity of a number of protein phosphatases, such as protein tyrosine phosphatase, protein phosphatase 2A and calcineurin [48].

### 2.4. Oxidative Stress

As shown above, ROS under normal and controlled conditions mediate and regulate physiological functions of the body. However, ROS over-accumulation caused by losing the balance between the generation and elimination of ROS results in severe deleterious effects to the cells, organs and body, a phenomenon known as oxidative stress. Oxidative stress can result from over generation of ROS in various conditions, such as injury, inflammation, aging, chronic diseases, *etc.* Alternatively, ROS accumulation and oxidative stress could be due to the diminished abilities in the elimination of ROS. For example, reduction in the level of GSH, an important intracellular antioxidant, causes redox imbalance in many disorders, such as in Parkinson’s diseases, HIV infection, liver disease, and cystic fibrosis, *etc.* [51–53].

Free radicals can pass freely through cell and nucleus membranes, and oxidize biomacromolecules. Lipid peroxidation caused by ROS leads to membrane leakage [54]. The oxidation of amino acid residues (especially cysteine residues) results in the formation of protein-protein cross-links, leading to dysfunction of these proteins. In addition, oxidation of kinase and phosphatase dysregulates the signal pathways as well. ROS-induced DNA peroxidation interrupts gene transcription and causes gene mutations. The principal oxidative DNA damage (ODD) products include 8-hydroxyadenine (8-OH-Ade), 8-hydroxyguanine (8-OH-Gua; and its deoxynucleoside equivalent, 8-OH-dG), 5,6-dihydroxy-5,6-dihydrothymine (thymine glycol, Tg) and ring-opened lesions: 4,6-diamino-5-formamidopyrimidine (FapyAde) and 2,6-diamino-4-hydroxy-5-formamidopyrimidine (FapyGua) [55]. The oxidant-damaged DNA leads to gene mutations, microsatellite instability, and effects on transcription factor binding [56]. RNA may be more vulnerable to oxidative insults than DNA given its generally single-stranded state and accessibility to the oxidant-producing mitochondria. The most commonly quantified nucleotide adducts include 8-hydroxyguanine (8-OHG), 8-hydroxyadenine (8-OHA), 5-hydroxycytosine (5-OHC), 2,6-diamino-4-hydroxy-5-formamidopyrimidine (fapyguanine), and 4,6-diamino-5-formamidopyrimidine (fapyadenine) [57], all of which are found in both DNA and RNA. Oxidative damage to protein-coding RNA or non-coding RNA will, in turn, potentially cause errors in proteins and/or dysregulation of gene expression [58]. As a consequence of peroxidations of lipids, proteins, RNA and DNA, high levels of ROS cause damage to various cellular components and ultimately result in cell death. Excessive ROS result in a number of chronic diseases typified by neurodegenerative diseases and also mediate therapeutic side effects, such as chemotherapy-induced cognitive impairment (or chemobrain).

## 3. ROS Mediates Neurodegenerative Diseases

### 3.1. Neurodegenerative Diseases

Neurodegenerative diseases are disorders in which the nervous system progressively and irreversibly deteriorates. Neurodegenerative diseases are commonly late-onset disorders, typified by Alzheimer’s disease (AD), Parkinson’s disease (PD), Huntington’s disease (HD) and Amyotrophic lateral sclerosis (ALS). AD is an age-dependent, chronic neurodegenerative disease, the leading cause of dementia amongst older people and the fourth most common cause of death in the Western world [59–61]. AD affects 4.5 million Americans, with 350,000 new diagnoses made each year [22,60]. AD is second only to cancer as the most costly disease in the United States, with both direct and indirect health care expenses costing approximately $100 billion/year [60]. A hallmark of AD pathology is brain atrophy, subsequent to gradual cell loss in the CNS. Characteristic neurofibrillary tangles and neural plaques are seen post mortem. PD is another CNS neurodegenerative disease afflicting millions of the older population, with most cases occurring after the age of 50. Early on in the course of the disease, the most obvious symptoms are movement-related, including tremor, rigidity, slowness of movement and difficulty with walking and gait. The motor symptoms of PD result from the slow degeneration of dopamine-generating neurons in the substantia nigra of the basal ganglia, a region of the midbrain, leading to progressive loss of muscular co-ordination and balance [62]. Later, thinking and behavioral problems may arise, with dementia commonly occurring in the advanced stages of the disease, whereas depression is the most common psychiatric symptom. Other symptoms include sensory, sleep and emotional problems. HD characterized with abnormal involuntary writhing movements called chorea is a neurodegenerative genetic disorder that affects muscle coordination and leads to cognitive decline and psychiatric problems. Although physical symptoms of HD can begin at any age from infancy to old age, it usually begins between 35 and 44 years of age. Medical imaging techniques such as computerized tomography (CT) and magnetic resonance imaging (MRI) can show atrophy of the caudate nuclei and striatal volume in the disease [63,64]. ALS is a fatal chronic neurodegenerative disease with a prevalence of 1–2 per 100,000 [65]. Approximately 90%–95% of ALS cases are sporadic ALS, with familial ALS (or inherited) comprising the remaining 5%–10% of cases. In ALS pathogenesis, it has been assumed that damage to motor neurons in the primary motor cortex, corticospinal tracts, brain stem, and spinal cord leads to the muscle weakness that typifies ALS.

### 3.2. Aggregation of Misfolded Proteins as the Hallmark of Neurodegenerative Diseases

Neurodegenerative diseases such as AD, PD, HD and ALS share a similarity in aggregation of disease-specific proteins in the CNS. Protein misfolding occurs in AD, manifested by accumulation of abnormally folded beta-amyloid (Aβ) and tau proteins in the brain [66]. Under normal conditions, a transmembrane protein amyloid-β precursor protein (AβPP) is critical to neuron growth, survival and post-injury repair [67,68]. However, in AD, APP is cleaved into a small peptide, 39–43 amino acids in length, called Aβ, by enzymes through proteolysis [69]. Aβ forms clumps and deposits outside neurons in dense formations known as senile plaques [70,71]. In addition, phosphorylation of Tau protein results in abnormal aggregation and dysfunction of this protein in AD. In PD, the protein alpha-synuclein (αSyn) binds to ubiquitin and forms proteinaceous cytoplasmic inclusions, named Lewy bodies. Over accumulation and posttranslational modification of αSyn results in death of dopaminergic neurons [72,73]. All humans have two copies of the *huntingtin* gene (*htt*), which codes for the protein huntingtin (Htt). The mutant huntingtin protein (mHtt) is an aggregate-prone protein. During the natural clearance process of cells, these proteins are retrogradely transported to the cell body for destruction by lysosomes. Under pathological conditions, these mutant proteins aggregate and damage the retrograde transport of important molecules such as BDNF by damaging molecular motors as well as microtubules [74], causing pathological changes and disease symptoms. Prior to the destruction in ALS, motor neurons develop proteinaceous inclusions in their cell bodies and axons. These inclusions often contain ubiquitin, and generally incorporate one of the ALS-associated proteins: SOD1, TAR DNA binding protein (TDP-43, or TARDBP) or FUS. Protein degradation pathways play a crucial role in removing misfolded proteins and preventing protein aggregation. Accumulation of ALS-specific proteinaceous inclusions may be partly due to defects in protein degradation [75].

### 3.3. Involvement of ROS in Misfolded Protein Formation

**Figure 3.**
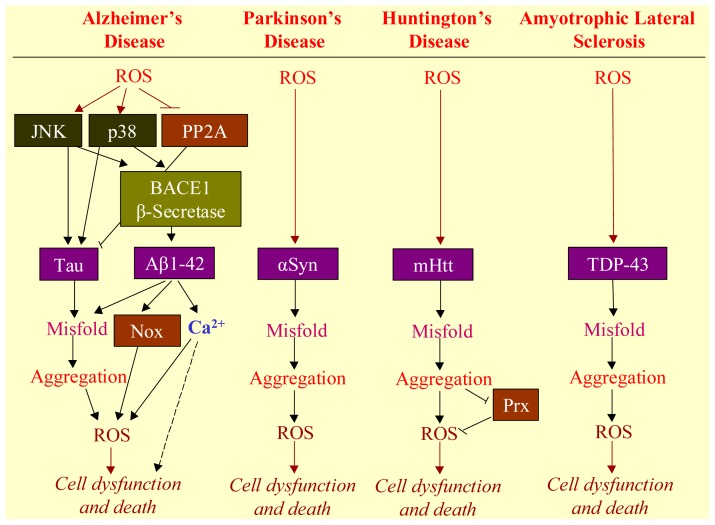
The causal relationship between ROS and misfolded proteins underlying neurodegenerative diseases. A common feature in neurodegenerative diseases is the existence of hallmark protein(s) for each, such as Tau and beta-amyloid (Aβ) for Alzheimer’s disease (AD), alpha-synuclein (αSyn) for Parkinson’s disease, mutant huntingtin protein (mHtt) for Huntington’s disease, and TAR DNA binding protein (TDP-43) for Amyotrophic lateral sclerosis. ROS mediate neurotoxicity in each of these diseases through modifying the hallmark protein by oxidation. In AD, ROS could also activate c-Jun *N*-terminal kinases (JNK) and p38, and deactivate protein phosphatase 2A (PP2A). JNK and p38 promote the expression of Tau, which is inhibited by PP2A. The activation of JNK and p38 further stimulate AβPP cleaving enzyme 1 (BACE1), causing Aβ1-42 accumulation, which leads to activation of NADPH oxidase (Nox) to produce additional O_2_^•−^, and results in Ca^2+^ influx to elicit excitatory neurotoxicity. In Huntington’s disease, aggregation of mHtt could inhibit peroxiredoxin (Prx), which is an antioxidant.

Intracellular neurofibrillary tangles (NFT) and extracellular deposits of Aβ are the two major histopathological hallmarks of AD. NFT are composed of bundles of paired helical filaments (PHF), the major component of which is the microtubule-associated protein Tau. Tau hyperphosphorylation appears to be a critical event leading to abnormal aggregation and disrupted function of this protein in the affected neurons in AD. ROS are actively involved in Tau phosphorylation. In an *in vitro* model of chronic mild oxidative stress where heme oxygenase 1 (HO-1) is induced without neuronal cell death, ROS were found to phosphorylate Tau at the serine 399/404 epitope of PHF-1 in a time-dependent manner [76]. Acrolein, a peroxidation product from arachidonic acid, increases the phosphorylation of Tau at the site recognized by antibody to PHF-1 both in human neuroblastoma cells and in primary cultures of mouse embryo cortical neurons [77]. In addition, increased activities of c-Jun *N*-terminal kinases (JNK) and p38 and decreased activity of PP2A under chronic oxidative stress condition are likely involved in tau phosphorylation as well [54] (Figure 3).

Once phosphorylated, Tau and other cytoskeletal proteins are vulnerable to modification by carbonyl products of oxidative stress [78,79] and consequent aggregation into fibrils [78]. Modifications of Tau by 4-hydroxy-2-nonenal (HNE) are found to promote and contribute to the generation of the major conformational properties defining neurofibrillary tangles [80].

Aβ found in senile plaques is considered to have a causal role in AD. H_2_O_2_ at 100–250 μM results in an increase in the levels of intracellular Aβ in human neuroblastoma SH-SY5Y cells [81]. Oxidative stress results in accumulation of potentially neurotoxic Aβ peptide by inducing the amyloidogenic process of AβPP and increasing the activity of β-secretase [82,83]. Cerebral amyloid angiopathy is associated with most cases of AD, characterized by Aβ deposits in brain vessels [84]. Oxidative stress is found triggering the amyloidogenic pathway in human vascular smooth muscle cells by up-regulation of AβPP cleaving enzyme 1 (BACE1) expression and secretion of Aβ1–40 and Aβ1–42 with mediation of c-Jun *N*-terminal Kinase and p38 MAPK [85].

Oxidative stress actively regulates protein aggregation in PD. Posttranslational modifications of the αSyn by oxidative stress, including those by 4-hydroxy-2-nonenal (HNE-αSyn), nitration (n-αSyn), and oxidation (o-αSyn), have been implicated in promotion of oligomerization of αSyn. Among them, HNE-αSyn and n-αSyn are more prone to forming oligomers than unmodified αSyn. The cellular toxicity of HNE-αSyn is significantly higher than other postranslationally modified species [72].

Aggregation of mHtt plays an important role in the pathogenesis of HD. The mHtt can aggregate at distinctive conformations that have different neurotoxicity, and different conformations of mHtt exist in different brain regions in HD mice [86]. Oxidative modification of the aggregated mHtt facilitates an increase in the size of aggregates and changes the conformation of aggregated mHtt [87]. Oxidative stimuli have been found to enhance the polyglutamine-expanded truncated *N*-terminal Huntingtin aggregation and mHtt-induced cell death [88]. In addition, aldolase C, aconitase, GFAP, tubulin, peroxiredoxin (Prx) 1/2/6, glutathione peroxidase and aB-crystallin have been demonstrated as targets of oxidative modification in HD [89].

TDP-43 is the major disease protein in ubiquitin-positive inclusions of ALS. Accumulation of insoluble TDP-43 aggregates could impair normal TDP-43 function and initiate disease progression. Oxidation of cysteines in the RNA recognition motif of TDP-43 induces conformational changes that subsequently result in protein aggregation and loss of nucleic acid-binding activity to facilitate disease progression [90,91].

### 3.4. ROS-Bridging Misfolded Proteins and Cell Death

There is a causal relationship between ROS imbalance and misfolded proteins. In contrast to the phenomena, as described above, that oxidative stress results in generation and aggregation of misfolded proteins, misfolded proteins can also lead to excessive ROS causing neurotoxicity. Several lines of evidence have demonstrated that Aβ formation promotes ROS production. Aβ activates the prooxidative enzyme NADPH-dependent oxidase, leading to the production of O_2_^•−^[92]. Furthermore, H_2_O_2_ is directly generated during the process of Aβ aggregation [93]. Aβ can convert molecular oxygen into H_2_O_2_ by reducing divalent metal ions (Fe^2+^, Cu^2+^) [94,95]. Aβ-induced ROS accumulation results in lipid peroxidation and subsequent production of the cytotoxic HNE [96]. Aβ also elicits neurotoxicity by interfering with Ca^2+^ homeostasis. Aβ–induced HNE impairs membrane Ca^2+^ pumps and increases Ca^2+^ influx through voltage-dependent and ligand-gated calcium channels [97]. Alternatively, Aβ forms calcium-conducting pores in cell membranes [98,99]. Excessive intracellular calcium ([Ca^2+^]*_i_*) plays a central role in excitatory neurotoxicity. ROS also actively mediates Aβ–induced impairment in LTP.

LTP was impaired in wild-type hippocampal slices treated with exogenous Aβ1–42 and in slices from APP/PS1 mutant mice that model AD. MitoQ, a mitochondria-targeted antioxidant, and EUK134, a SOD and catalase mimetic, could prevent the impaired LTP [100]. In addition, in transgenic mice overexpressing SOD-2, Aβ-induced LTP impairments, superoxide generation and memory deficits are prevented [101,102].

Aggregation and neurotoxicity of misfolded αSyn, as mentioned above, are crucial mechanisms for progressive dopaminergic neurodegeneration associated with PD. In addition, the cellular toxicity of HNE-αSyn was significantly higher than other posttranslationally modified species. Exposure of dopaminergic neurons to HNE-αSyn has been found to trigger the production of intracellular ROS, preceding neuronal cell death. Antioxidant treatment effectively protected cells from the damage triggered by HNE-αSyn [72]. ROS have been also revealed to mediate misfolding and aggregation of mHtt–induced neurotoxicity and mitochondria in mHtt PC12 cells generate high level of ROS. On the other hand, mHtt decreases the expression of the antioxidant protein Prx 1, while overexpression of wildtype Prx1 suppresses mHtt-induced toxicity [103]. Various missense mutations of TDP-43 are identified in patients with ALS. ROS also mediates TDP-43-induced neurotoxicity. TDP-43-expressing cells displayed markedly increased markers of oxidative stress, apoptosis, and necrosis in yeast [104]. In a motor neuron-like cell system that was stable-transfected with wild type and mutant TDP-43, mutant TDP-43 induced mitochondrial dysfunction and oxidative damage that was indicated by an increase in lipid peroxidation [105].

Another way for ROS to bridge misfolded protein to cell death is its effect on the intracelluar and/or extracellular clearance capability for aberrant proteins. The main intracellular pathways for the degradation and recycling of proteins are the ubiquitin/proteasome system (UPS) and the autophagy-lysosomal pathway. Under a pathological condition, such as in PD, the ubiquitin-proteasome system and mitophagy are impacted by oxidative stress so that a decreased clearance rate results in the accumulation of alpha-synuclein [106,107]. Extracellular clearance pathways include interaction of neurons with astrocytes and microglia [108]. Oxidative stress can result in microglial senescence in response to intracellular accumulation of iron. The senescent microglia reduce the ability in clearance of aberrant proteins, such as ApoE, α-synuclein, and Aβ42, *etc* [109]. For example, the projections of microglia from aged mice, which surround Aβ fibrillary aggregates, showed a diminished ability to internalize Aβ peptide. Thus, reduced clearance of Aβ42 would contribute to the pathogenesis of AD, a late-onset disease.

Although there is a great deal of scientific literature that describes the debilitating effects of ROS, oxidative stress may not readily contribute directly to cell death. For example, a downstream response of oxidative stress-caused misfolded proteins, named ER stress, has recently attracted extensive attention for its role in cell death.

### 3.5. ER Stress Bridging Misfolded Proteins and Cell Death

The ER is an organelle that crucially controls, in addition to calcium and redox homeostasis, protein synthesis, folding and trafficking. Protein folding is fulfilled in the lumen of the ER where related proteins and enzymes are located, including immunoglobulin binding protein (BiP), GRP94, protein disulfide isomerase (PDI), calnexin, calreticulin, *etc*. [110]. Only properly folded proteins can export to the Golgi apparatus for further modification. In contrast, misfolded or incompletely folded proteins are retained in the ER, leading to a cell adaptive response, named the unfolded protein response (UPR) or ER stress response.

ER stress is a physiological adaption to accumulated folded protein in the ER by attenuation of protein synthesis to reduce protein load, transcriptional induction of ER chaperone genes to accelerate protein folding, and degradation of misfolded proteins by the ER-associated degradation (ERAD) machinery to remove these proteins [111]. However, if ER stress is prolonged or excessive in a disturbance, such as hypoxia, glucose deprivation or oxidative stress, the cells will elicit apoptotic processes to remove over-stressed cells. Three ER stress sensors have been reported initiating UPR; these include inositol requiring enzyme-1 (IRE1), protein kinase RNA-like ER kinase (PERK) and activating transcription factor 6 (ATF6). ATF6 promotes folding of protein and removal of misfolded protein by upregulation of chaperones, foldases, and components of the ERAD machinery, while IRE1 and PERK are functionally involved in both reducing ER protein load and triggering apoptosis [112]. The key role of ER stress in mediation of neurodegenerative diseases has been well documented recently [110,113].

## 4. ROS Mediated Chemotherapy-Induced Neuropathy

In addition to the ROS-mediated neurodegenerative diseases, ROS also mediates chemotherapy-induced neurotoxicity in both the central and peripheral nervous systems. Side effects of anticancer drugs in the CNS and PNS can sometimes be too severe to continue chemotherapy treatment for cancer patients.

### 4.1. The Role of ROS in Chemobrain

Chemotherapy has improved survival rates in patients with many of the common cancers. However, one of the most common complications of chemotherapeutic drugs is toxicity to the CNS, termed chemotherapy-induced cognitive impairment, chemotherapy-induced cognitive dysfunction, post-chemotherapy cognitive impairment (PCCI), chemo fog, or chemobrain. Chemobrain can be very frustrating both for those who are living with cancer, and their loved ones who are trying to support them. Chemobrain can seriously affect quality of life and life itself in cancer patients. This toxicity can manifest itself in many ways, including encephalopathy syndromes and confusional states, seizure activity, headache, cerebrovascular complications and stroke, visual loss, cerebellar dysfunction, and spinal cord damage with myelopathy [114]. There is reliable evidence that, as a result of treatment, a subset of cancer survivors experience cognitive problems that can last for many years following the completion of chemotherapy. These include attention deficits, memory loss, and confused thought processes. Up to 70% of patients with cancer report that these cognitive difficulties persist well beyond the duration of treatment [115–117]. Studies that have measured cognitive function using standardized neuropsychological assessments have found mild to moderate effects of chemotherapy on cognitive performance in 15%–50% of the survivors after treatment [118,119]. Longitudinal studies have shown that, in a subset of survivors, cognitive difficulties can persist for between 1 and 2 years following the completion of chemotherapy [120,121]. Cross-sectional studies have found cognitive impairments lasting between 4 and 10 years following chemotherapy [122,123]. Although recent prospective studies show that ~20% of cancer patients experience cognitive dysfunction even before chemotherapy [124,125], chemotherapy agents produce significant cognitive impairment in laboratory animals that are free from cancer as well as from other treatment- and diagnosis-related factors. Healthy rodents that are given chemotherapy show increase in cell death in the CNS [126], increase in oxidative stress [126,127], increase in microglia activity [128], suppression of hippocampal neurogenesis [129], decreases in levels of neurotrophic factors [130], and decreases in levels of hippocampal catecholamines [131], as compared to baseline values. The etiology of chemotherapy-induced cognitive impairment is largely unknown, but several candidate mechanisms have been suggested, including oxidative stress, impaired blood-brain barrier (BBB), neuroinflammation, and decreased neurogenesis, *etc* [132].

**Figure 4.**
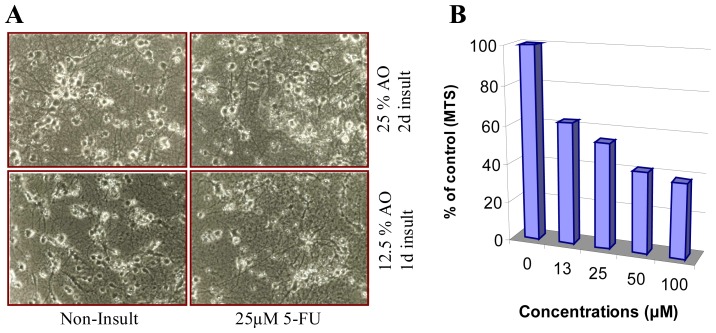
Effect of Antioxidants (AO) on the Neurotoxicity of 5-Fluorouracil (5-FU). (**A**) Morphological observation. Embryonic brain neurons were cultured for 13 days with concentration reduction of AO in culture medium to 25% and 12.5%, respectively. This process did not elicit cell death by itself (comparison of Non-Insult groups under higher and lower AO conditions). Treating neurons with 25 μM 5-FU did not cause observable neurotoxicity under 25% AO culture condition. However, 5-FU at the same concentration resulted in severe cell death when AO concentration was reduced to 12.5%, even for a shorter time periods. The cells were photographed under a phase-contrast microscope using a 10× objective; and (**B**) Quantification of cytotoxicity. 3-(4,5-Dimethylthiazol-2-yl)-5-(3-carboxymethoxyphenyl)-2-(4-sulfophenyl)-2*H*-tetrazolium, inner salt (MTS) was used for the quantitative assay, which directly indicates an enzymatic activity of mitochondria and indirectly reflects the survival cell number. The graph shows that the neurons under reduced AO condition were vulnerable to 5-FU. Data are expressed as mean (*n* = 6) percentage of Non-Insult groups (taken as 100%). 5-FU dose-dependently reduced MTS reading in reduced AO condition.

Oxidative stress plays a key role in cognitive disorders caused by certain types of anticancer drugs, such as antimetabolites, mitotic inhibitors, topoisomerase inhibitors and microtubule stabilizers, *etc*. [133]. These chemotherapeutic agents are not known to rely on oxidative mechanisms for their anticancer effects. Among the antimetabolite drugs, methotrexate (MTX), 5-fluorouracil (5-FU, a widely used chemotherapeutic agent), and cytosine arabinoside are most likely to cause CNS toxicity [114]. 5-FU can cause both acute and delayed neurotoxicity. Acute neurotoxicity manifests itself as encephalopathy cerebellar syndrome or as seizures. Acute neurotoxicity due to 5-FU is dose-related and generally self-limiting [134]. 5-FU readily crosses the blood-brain barrier and disrupts cell proliferation [130]. Clinically relevant concentrations of 5-FU are toxic for both CNS progenitor cells and non-dividing oligodendrocytes *in vitro* and *in vivo* [135]. Short-term systemic administration of 5-FU caused both acute CNS damage and a syndrome of progressively worsening delayed damage to myelinated tracts of the CNS associated with altered transcriptional regulation in oligodendrocytes and extensive myelin pathology [135]. Functional analysis also provided the first demonstration of delayed effects of chemotherapy on the latency of impulse conduction in the auditory system, offering the possibility of non-invasive analysis of myelin damage associated with cancer treatment [135]. Delayed neurotoxicity has been reported when fluorouracil was given in combination with levamisole; this form of subacute multifocal leukoencephalopathy is immune-mediated [134]. Although there is no report yet of 5-FU increasing CNS oxidative stress, it has been shown to induce apoptosis in rat cardiocytes through intracellular oxidative stress [136], to increase oxidative stress in the plasma of liver cancer patients [137], and to decrease glutathione in bone marrow cells [138]. Evidence for the involvement of ROS in 5-FU-induced neurotoxicity comes from our latest research, in which 5-FU elicits cell death of embryonic cerebral neurons only under the condition where antioxidants in the culture medium are reduced to a certain level (Figure 4). Another antimetabolite, MTX, can cross the blood-brain barrier as well [124]. It causes an increase of oxidative stress in cerebral spinal fluid and results in dysfunction of the CNS in MTX-treated patients with pediatric acute lymphoblastic leukemia [139,140]. A recent observation also indicates that genetic polymorphism for methionine is a potent risk factor for MTX-induced CNS toxicity [141].

### 4.2. The Role of ROS in Chemotheray-Induced Peripheral Neuropathy (CIPN)

CIPN is one of the most common and serious side effects of chemotherapy, and it can result in dose reductions or early discontinuation of chemotherapy, which reduces the efficacy of cancer treatments. It can cause debilitating symptoms and also significantly impacts the patient’s quality of life. An estimated 30 to 40 percent of cancer patients treated with chemotherapy experience CIPN [142].

The peripheral nervous system (PNS) consists of sensory neurons running from stimulus receptors that inform the CNS of the stimuli, and motor neurons running from the spinal cord to the effectors that take action. In CIPN, an anticancer drug could impair both sensory and motor functions. The symptoms usually start in the hands and/or feet and creep up the arms and legs. Sometimes it feels like a tingling or numbness; other times, it’s more of a shooting and/or burning pain or sensitivity to temperature. It can include sharp, stabbing pain. It can also cause hearing loss, blurred vision and/or change in taste [143–145]. CIPN can make it difficult to perform normal day-to-day tasks like buttoning a shirt, sorting coins in a purse, or walking. In addition, the motor neuron dysfunction manifests itself as cramps, difficulty with fine motor activities (e.g., writing or dialing a phone), gait disturbances, paralysis, spasms, tremors and weakness.

The chemotherapeutic drugs that most commonly elicit CIPN include platinum compounds (cisplatin, carboplatin, oxaliplatin), vincristine, taxanes (docetaxel, paclitaxel), epothilones (ixabepilone), bortezomib (Velcade), thalidomide (Thalomid) and lenalidomide. Many other chemotherapeutic agents, such as Ixabepilone, arsenic trioxide, cytarabine, etoposide, hexamethylmelamine, Ifosfamide, methotrexate, and procarbazine can also induce CIPN.

The underlying causes for CIPN on the cellular and tissue level are still largely a matter of speculation. Oxidative stress may play a key role in CIPN. It was found that antioxidant machinery (e.g., plasma glutathione (GSH) and α- and γ-tocopherol concentrations) of cancer patents with chemotherapy was decreased and that the GSH redox state became more oxidized [146]. Oxidative stress was found as an important mediator in a rat model of painful oxaliplatin-induced neuropathy, since the increases of carbonylated protein and thiobarbituric acid reactive substances in the plasma of oxaliplatin-treated rats were indicative of the resultant protein oxidation and lipoperoxidation, respectively. The same pattern of oxidation was also revealed in the sciatic nerve and the spinal cord where the damage reached the DNA level [147]. In addition, oxidative imbalance was also indicated to mediate inflammatory pain [148]. The anticancer drug cisplatin results in severe cell death of sensory neurons derived from dorsal root ganglia when apurinic/apyrimidinic endonuclease/redox factor-1 that is involved in DNA base excision repair of oxidative DNA damage and in redox regulation of a number of transcription factors is suppressed [149]. Oxidative stress was also found to impair the autonomic nervous system, such as hearing loss [150,151].

## 5. Antioxidation Is a Strategy to Block the Progress of Neurodegenerative Diseases and Chemotherapy-Induced Chemobrain and Peripheral Neuropathy

### 5.1. Antioxidative Treatment for Neurodegenerative Diseases

Since ROS mediates neurotoxicity in a number of neurodegenerative disorders, one strategy in disease control has been focused on development of antioxidants as preventive and therapeutic molecules. These include vitamin C, vitamin E, glutathione, coenzyme Q (CoQ), carotenoids, and melatonin, *etc* [152]. For example, vitamin E supplementation in the AD mouse model demonstrated a reduction in Aβ deposition and improved cognition [153]. These changes were specifically effective in young AD mice [154]. Melatonin has been shown to positively affect memory performance and provide protection against oxidative stress-induced damage in all of the animal AD models [141]. CoQ10 (ubiquinone) is a potent antioxidant. A study revealed that CoQ10 scavenges peroxy radicals faster than α-tocopherol [155]. A co-administration of CoQ10 with creatine was recently shown to provide additive neuroprotective effects in models of Parkinson’s and Huntington’s diseases by blocking impairment of glutathione homeostasis and reducing lipid peroxidation and DNA oxidative damage in the striatum [156]. For treatment of ALS, many antioxidants have been developed in G93A mice, including manganese porphyrin, rasagiline, DP109 and 460, M30-HLA20, NAC and vitamin E [157]. Among them, rasagiline and DP109 and 460 have been found to improve motor performance and nearly all of them improve survival.

### 5.2. Antioxidative Treatment for Treatment of Chemobrain and CIPN

Cisplatin is a platinum-based chemotherapeutic agent widely used for the treatment of various types of cancer. Cisplatin is the most common drug to cause severe CIPN. In addition, patients receiving cisplatin treatment often suffer from chemobrain as well. There is no cure for this side effect. In laboratory studies, a sulfur-containing nucleophilic antioxidant d-Methionine (d-Met) has been shown to prevent cisplatin-induced neurotoxicity in cultured CNS neurons [158] and animals without antitumor interference [159,160]. Mirtazapine, a less-known antioxidant, has recently also shown chemoprotective effects against cisplatin-induced oxidative stress and DNA damage in the rat brain [161].

The antineuropathic effect of antioxidant silibinin or α-tocopherol reduces oxaliplatin-induced behavioral alterations by about 50% [147]. Short-term systemic treatment with α-lipoic acid (an antioxidant) could completely prevent hypersensitivity if administered prior to the anticancer drug bortezomib or oxaliplatin [162]. For prevention of the onset of CIPN, further clinical testing of many antioxidative stress agents, such as glutathione, acetyl-l-carnitine and alpha-lipoic acid was suggested [163].

## 6. Methods to Determine Cellular Redox Status and Screen Antioxidants

For a convenient experimental research reference for readers, in this section, we review methods that have been used in the measurement of cellular redox status, which will be useful for drug evaluation as well. As we know, the redox status of cells is dependent upon the balance between ROS/RNS generation and antioxidant strength. Oxidative stress not only occurs through the overproduction of ROS/RNS but also under a case of a depleted or weaken antioxidant system, for example the depletion of glutathione, in the cell. Therefore, the redox status of cells could be determined by a method to measure intracellular or intramitochondrial ROS/RNS accumulation and/or, in some cases, the strength of antioxidant systems.

### 6.1. Methods to Evaluate the Involvement of ROS and/or RNS in Physiological or Pathophysiological Processes

A simple way to understand the possibility for ROS or RNS to be involved in a bio- or pathophysiological process is application of antioxidants or a NOS inhibitor to block the event.

Various synthetic chemicals with clear antioxidant activity have been developed and used for this purpose. These include Zinc chloride, *N*-acetyl-l-cysteine, 1,10-phenanthroline and pyrrolidine [164–166]. In addition, natural products such as vitamin C, vitamine E, melatonin, *etc* have also been utilized for blockage of ROS-mediated neurotoxicity [167–169].

2-(4-Carboxyphenyl)-4,4,5,5-tetramethylimidazoline-1-oxyl-3-oxide (c-PTIO) and the flavonoid quercetin are specific NO scavengers [170–172]; Uric acid and hesperetin are peroxynitrite scavengers [173,174]. These have been used for determination of RNS involvement as well.

Blockage of a process with a reagent against ROS or RNS alone is not sufficient to confirm the involvement of ROS or RNS, since target specificity of the agent and complexity of signaling pathways in the cells all affect outcome of the assay. To verify the result and reveal the actual mechanisms, ROS or RNS should be measured directly using the following methods.

### 6.2. Methods to Determine ROS and RNS Generations

There are different methods to measure ROS and RNS an each has advantages and disadvantages. Therefore, the method selected by a researcher is dependent on the experimental purpose. Since there are no definitively superior parameters, a combination of methods is encouraged.

#### 6.2.1. Fluorescent and Chemiluminescent Probes for ROS

The most convenient way to determine intracellular or intramitochondrial ROS accumulation is the utilization of fluorescent or chemiluminescent probes. The probes used for this purpose are cell permeable and the measurements are generally based on the oxidative activity of ROS.

2′,7′-Dichlorodihydrofluorescein diacetate (DCFH-DA) is commonly used to measure intracellular ROS level. DCF-DA enters cells and accumulates mostly in the cytosol. Cellular esterases cleave DCFH-DA into 2′,7′-dichlorodihydrofluorescein (DCFH_2_), which is further oxidized, in the presence of H_2_O_2_, by peroxidases, cytochrome c and Fe^2+^ into 2′,7′-dichlorofluorescein (DCF). The fluorescent strength of DCF in the cytosol can be measured at 530 nm using a fluorescent plate reader when the sample is excited at 485 nm. The fluorescence at 530 nm can also be measured by a flow cytometer [175,176]. Since NO, and peroxynitrite also oxidize DCFH_2_[177,178], the fluorescent strength from DCF reflects not only the H_2_O_2_ level in the cytosol. Furthermore, DCF is membrane permeable and can leak out of cells; to avoid this, other probes have been developed. For example, dihydrocalcein can freely pass cell membranes, being oxidized to a charged green-fluorescent calcein, which has excellent retention properties [179].

Dihydrorhodamine 123 (DHR) is red fluorescent dye to measure the ROS in the mitochondria. DHR is oxidized to rhodamine 123; the latter is lipophilic and positively charged, which is retained in mitochondria by the membrane potential. In this way, accumulated rhodamine 123 is measured around 536 nm when the sample is excited at 500 nm. However, this probe showed sensitivity to ^•^OH, ONOO^−^, O_2_^•^, but was less responsive to O_2_^•−^, H_2_O_2_ and NO^•^[180].

Dihydroethidium or dihydroethidine (DHE) and its derivative mitoSOX are fluorescent probes to detect O_2_^•−^ in the cells and mitochondria, respectively [181–183]. O_2_^•−^ reacts with DHE to form 2-hydroxyethidium (2-OH-E+), which shows blue fluorescence at 420nm when the sample is excited at 370nm. The mitoSOX shows red fluorescence at 580 nm when the sample is excited at 510 nm. The limitation for this method is that another fluorescent oxidation product ethidium (E+) significantly contributes to the total fluorescence intensity, and fluorescent spectra between 2-OH-E+ and E+ are overlapped. Thus, the use of fluorescence-based techniques alone to measure O_2_^•−^ can be very misleading [184].

Luminol and lucigenin chemiluminescent probes are alternative, cell permeable means for measurement of ROS level. The luminol assay is sensitive to H_2_O_2_, and its analogue L-012 may be more sensitive than luminol to O_2_^•−^ and ONOO^−^[185], and lucigenin could be more sensitive than luminal to O_2_^•−^ as well [186,187]. However, the reliability of these assays should be checked since redox cycling of luminol or lucigenin can lead to extra O_2_^•−^ production [186,188]. If O_2_^•−^ level is under a threshold concentration, lucigenin may still be a valid probe for detecting O_2_^•−^ production through enzymatic and cellular sources [189].

#### 6.2.2. Chromatography without or with Mass Spectrometry

As mentioned above, DHE-based fluorescent analysis is not suitable for quantification of intracellular O_2_^•−^. In contrast, the product 2-OH-E+ from the reaction of O_2_^•−^ and fluorescent dye DHE can be easily distinguished from E+, peaking at elution time, by HPLC [190].

#### 6.2.3. Electron Paramagnetic Resonance with Spin Trap Technique

Spin trapping-assisted electron spin resonance (ESR) perhaps is a technique to directly detect free radials. The short lifetime of free radicals makes it difficult to detect with direct electron spin resonance (ESR) in room temperature. Spin trapping provides a means, in principle, to overcome this problem. In this approach, addition of radicals to a nitrone spin trap results in the formation of a stable spin adduct of the radical, and the spin adduct yields a distinctive spectrum by ESR measurement. The most commonly used spin trap in study of O_2_^•−^ and ^•^OH is 5,5-dimethyl-pyrroline N-oxide (DMPO) [191,192]. Recently, this technique has also been used for *in vivo* studies [193,194]. However, the major limitation for this technique is the rapid removal of radical adduct *in vitro* and *in vivo* by enzymic metabolism and by direct reduction with antioxidants [195]. Besides, ESR facility is not available to majority of laboratories.

#### 6.2.4. Fluorescent and Chemiluminescent Probes for RNS

As mentioned above, intracellular NO and peroxynitrite can be determined with DCFH-DA. The other probes for RNS are listed as follows:

4,5-Diaminofluorescein diacetate (DAF2-DA) is a fluorescent method to determine NO. In the cells, DAF2-DA reacts with NO in the presence of oxygen to yield the highly fluorescent triazolofluorescein (DAF-2T), which can be fluorometrically measured at 538 nm, showing green color, when a sample is excited at 485 nm [196,197]. With high cell permeability, the DAF2-DA method allows monitoring dynamical change of fluorescence in the cells, a common feature of fluorescent probes. Since increased intracellular free calcium is suggested to unspecifically affect the fluorescent strength of this probe [198], caution must be used with result interpretation, and use of another method is recommended to confirm NO strength.

Several fluorescence-based methods to detect ONOO^−^ have been developed by several groups, but are not widely used so far. It is too early to judge their advantages and disadvantages. These include NiSPY probes [199] and boronate probes [200]. The latter may provide more reliable method than DCFH-DA in detection of ONOO^−^ strength [183].

#### 6.2.5. Activity of Enzymes in RNS Generation

As mentioned above, NOS catalyze NO formation. Therefore, the activity of NOS is another indicator for cellular nitrosative stress. NOS convert l-arginine to l-citrulline and NO at a 1:1 ratio in stoichiometry. Thus, measurement of l-citrulline is used as a NOS activity assay [201]. l-Citrullin is separated from l-arginine by an acidic cation exchanger [202]. This assay is used to determine mtNOS activity and its regulation [203]. Since the citrullin could be produced through arginase, that path may need to be blocked in some circumstances, such as testing liver extract where high-level arginase exists.

### 6.3. Methods to Measure the Strength of Antioxidant Systems

#### 6.3.1. Fluorescent Probes for Glutathione

As mentioned above, glutathione is an important antioxidant system. Under certain circumstances, such as in the onset of apoptosis, glutathione could efflux from the cells [204], which lowers the reducing capability of the cells and can consequently result in oxidative stress without interference of ROS generation. Therefore, GSH depletion is often taken as a marker of oxidative stress [205].

Monochlorobimances and monobromobimane are membrane permeable probes. The probes can bind to sulfydryl groups and generate fluorescence [206], which can be measured around 461–490 nm, when the sample is excited at 380–395 nm.

Ortho-phthaldialdehyde (OPT) is another fluorescent probe to assess total glutathione levels in protein precipitated cell lysate [207]. The thiol adduct is fluorometrically measured at 455 nm when the sample is excited at 334 nm.

5,5-Dithiobis (2-nitrobenzoic acid) (DTNB) is the fluorescent probe to assess glutathione level, which is measured at 420 nm when the sample is excited at 350 nm [208,209].

Since all probes for determination of glutathione react with protein sulfhydryl residues of protein, it is recommended to determine GSH level in a protein-free condition.

#### 6.3.2. Activity Measurement of Enzymatic Antioxidants

As mentioned above, enzymes SOD, glutathione peroxidase (GPx), thioredoxin reductase (TR) and catalase (Cat) are involved in cell defense by converting ROS to nontoxic metabolites. The levels and activities of these enzymes are due to the change under abnormal conditions. The levels of these enzymes could be determined with immunoassays, such as immunocytochemistry, flow cytometry, Western blot, *etc.* The expressions of the enzymes can be measured with RT-PCR and Northern blot. The following focuses on activity analysis of these enzymes. The assays can commonly be used to measure an enzymatic activity in plasma, serum, erythrocyte lysates, tissue homogenates, and cell lysates.

The superoxide dismutase (SOD) assay is a method to measure activity of SOD. In this assay, a water soluble tetrazolium salt WST-1 (2-(4-iodophenyl)-3-(4-nitrophenyl)-5-(2,4-disulfophenyl)-2*H*-tetrazolium, monosodium salt) is converted to a formazan dye WST-1 formazan by O_2_^•−^, which is measurable at 450 nm [210]. SOD reduces the level of O_2_^•−^ by dismutation of it into H_2_O_2_ and O_2_, and thereby lowers the rate of WST-1 formazan. In contrast, any reduction in SOD activity will result in an increase in the signal of WST-1 formazan. This assay has been used to measure ROS activity under many different conditions, such as in amyloid precursor protein transgenic mice and diabetic mouse models [211,212].

Glutathione reductase (GR) assay is an approach to determine activity of GR. GR is a ubiquitous enzyme that catalyzes the reduction of oxidized glutathione (GSSG) to glutathione (GSH), which is essential for maintenance of adequate levels of reduced cellular GSH for antioxidant reaction. This assay utilizes enzymatic-recycling method for measurement of activity of glutathione reductase. The yield of glutathione interacts with DTNB and generated a yellow-coloreed 5-thio-2-nitrobenzoic acid (TNB), which is absorbed at 405–412 nm. The activity of glutathione reductase could be suppressed in certain abnormal conditions, such as from side effects of therapeutic drugs [213], Alzheimer’s disease [214,215], and age-related macular degeneration [216].

Glutathione peroxidase (GPx) assay is used to judge activity of GPx. GPx is one class of the most important enzymes for detoxification of peroxides and prevention of lipid peroxidation in living cells. It reduces lipid hydroperoxides to their corresponding alcohols and catalyzes the reduction of H_2_O_2_ to H_2_O while converting GSH to GSSG. GSSG is reduced back to GSH by glutathione reductase. In this process, NADPH is oxidized to NADP, which has absorbency at 340 nm with a spectrophotometer [217,218]. Thus, this assay is used to indirectly measure the activity of GP. However, the activity of GR theoretically affects the outcome of this assay since it is involved in the reactions.

Catalase (CAT) assay is a way to measure activity of CAT. Catalase is a ubiquitous antioxidant enzyme that is present in nearly all living organisms. It functions to catalyze the decomposition of H_2_O_2_ to H_2_O and O_2_. The peroxidatic function of CAT can be used for determination of its enzyme activity. In the presence of an optimal concentration of H_2_O_2_, CAT reacts with methanol to form formaldehyde, which further reacts with 4-amino-3-hydrazino-5-mercapto-1,2,4-triazole (Purpald) to form a bicyclic heterocycle while the reaction solution changes from colorless to a purple color. The absorbance at 540 nm is spectrophotometrically read [219,220].

### 6.4. Method for Identification of Free Radical and Nitric Oxide Scavenger

#### 6.4.1. Method for Identification of Free Radical Scavenger

2,2-Diphenyl-1-picrylhydrazyl (DPPH) is a stable free-radical molecule. The DPPH radical with an odd electron shows a deep violet color in solution, since a strong absorption band is centered at about 517 or 520 nm. It becomes colorless or pale yellow when neutralized with antioxidants. In the cell-free system, this method is readily used to distinguish whether an agent that suppresses ROS in an *in vitro* or an *in vivo* system is a free radical scavenger or indirectly affect ROS level in the cells [167,221]. To understand how a test agent affects cell redox status, a functional study in a living system should be carried out.

#### 6.4.2. Method for Identification of Nitric Oxide Scavenger

This assay relies on a diazotization reaction that was originally described by Griess in 1879. The half-life if NO in biological systems ranges from ≤1 to around 30 s. However, NO in biological systems could be converted into nitrite ion (NO_2_^−^) and nitrate ion (NO_2_^+^). NO_2_^+^ can be reduced to NO_2_^−^ by nitrated reductase. The level of NO_2_^−^ can be measured with Griess reagents (see following information in text). In the cell-free assay system under a physiological pH, NO is spontaneously generated from sodium nitroprusside in aqueous solution. NO interacts with O_2_ to produce NO_2_^−^. Scavenger of NO, and when incubated with sodium nitroprusside at 250 °C for 150 min, competes with O_2_ leading to a decrease in NO_2_^−^ production. The samples from the above react with Griess reagents (1% sulphanilamide, 2% H3PO4 and 0.1% naphthyl ethylenediamine dihydrochloride) for 15–30 min. The absorbance of the red–pink chromophore formed during the diazotization of NO_2_^−^ is measured at 546 nm. The assay has been used for determination of NO scavenging activity of plant [222], biomolecules [223] and small molecules [224]. Although the Griess reaction is reliable, reproducible and convenient, and easily performed in most laboratories, the limit level of NO_2_^−^ for detection could be 2.5 μM, and the sensitivity is dependent on the sample used.

### 6.5. Method for Identification of Oxidized or Nitrated Large Molecules

#### 6.5.1. Method for Identification of Oxidized Large Molecules

In addition, HPLC methods have been widely used to measure ROS-oxidized large molecules. For example, HPLC has been successfully employed for measurement of lipid peroxidation products 4-hydroxynonenal (HNE) and malonaldehyde [225–227]. Immunocytochemistry assay is a convenient way to determine the existence of HNE under oxidative stress conditions [228,229]. HPLC is also utilized for determination of protein oxidation products generated by direct oxidation of amino acid residues or nitration of tyrosine [230,231]. In nuclear and mitochondrial DNA, 8-hydroxy-2′-deoxyguanosine (8-OHdG) or 8-oxo-7,8-dihydro-2′-deoxyguanosine (8-oxodG) is one of the predominant forms of free radical-induced oxidative lesions. HPLC with electrochemical detection is the most widely used method for quantitative analysis of these molecules [232]. RNA oxidation products 8-oxo-7,8-dihydroguanosine can be also determined using HPLC coupled to electrochemical detection [233]. However, the disadvantages of this method are inability to measure a free radical or oxidized large molecule in the cells, and the requirement of more samples to run.

#### 6.5.2. Method for Identification of Nitrated Large Molecules

The HPLC method has been also used to measure RNS-nitrated large molecules. NO and ONOO^−^ can nitrate guanine to form 8-nitroguianine, which exits in its free form or in its related nucleosides, nucleotides and DNA/RNA [234]. 8-nitroguianine in human urine can be quantified by HPLC-electrochemical detection (ECD) coupled with immunoaffinity purification [235]. Tyrosine can be nitrated to form 3-nitrotyrosine, which is found in its free form or within its related peptide and proteins [236]. Both nitrotyrosine and protein-associated 3-nitrotyrosine can be quantified with gas chromatography-tandem mass spectrometry (GC-tandem MS) and liquid chromatography-tandem mass spectrometry (LC-tandem MS) [237].

## 7. Concluding Remarks and Future Perspectives

Balance between generation and elimination of ROS is essential for maintenance of its physiological level and normal functions. ROS are generated at different locations in the cell, especially in the mitochondria. There are both enzymatic and non-enzymatic systems to maintain constant ROS levels. One way for normal levels of ROS to exert physiological function is through modulation of signaling pathways, such as activation of protein kinases and deactivation of phosphatases for LTP formation. Breaking the ROS balance has deleterious effects. Under low ROS condition, a living organism cannot carry out certain physiological functions. In contrast, high levels of ROS, either through over production or restricted elimination, puts a living organism under an oxidative stress condition.

Under oxidative stress, excessive ROS abnormally modify all biomacromolecules, causing lipid peroxidation, protein misfolding and aggregation, RNA mistranscription, and DNA damage and mutations. Among these damages, the role of ROS in the oxidation of hallmark proteins of neurodegenerative disease is of special interest, since this episode bridges non-specific cytotoxicity of ROS to a given neurodegenerative disorder. ROS seems to mediate and/or elicit neurotoxicity at least at dual levels—the regulation of signaling molecules and/or hallmark proteins by posttranslational modification and execution of damage to the cell under instructions of these specific molecules. Free radicals are capable to freely pass through the plasma membrane and result in membrane leakage and cell death. ROS damage accumulates following aging. When it is over the threshold, a late-onset disease occurs. Due to the high oxygen consumption of neurons, more ROS can accumulate in the brain compared to any other organs, and therefore results in neurodegenerative disease. The affected brain region and modified hallmark proteins by excessive ROS seem to determine the type of the disorder.

There is no doubt that development of antioxidants as neuroprotective drugs is a logical strategy. Although this approach is advisable, antioxidants alone have rarely shown beneficial effect for any of the neurodegenerative diseases. In fact, ROS are not the sole class of toxic factors to mediate neurotoxicity in a given neurodegenerative disease. Other factors, such as excitatory neurotoxicity, are also involved, which makes the underlying mechanism complicated. For efficient neuroprotection in humans, a drug with multiple drug targets or a combined administration of drugs with different drug targets is needed.

Compared with therapy, prevention of a late-onset disease is a top priority. Neurons are a terminally differentiated cell type, losing the capability of mitosis and proliferation. After neurons are lost in the brain, only stem cell-oriented cell therapy may shed a light for regional reconstruction. Therefore, prevention of neurodegenerative disease before severe nerve damage by control of the balance of ROS production and elimination is highly preferable. A balance of systems in living organisms is the best status for survival and health. This theory has been emphasized from both Eastern and Western civilizations in history, called “Taiji” or “Zhongyong” in China and “Golden mean” by Aristotle, respectively. In Taoism, everything has both yin and yang aspects, which are complementary to form a dynamic system as a whole, and Taiji is the balanced status. This is the basis for traditional Chinese medicine (TCM), where it is believed that the imbalance of systems in the human body is the reason for disease. Therefore, the correction of imbalance is an essential goal in TCM. This theory could be applied to prevention of late-onset diseases by controlling the ROS level.

In addition to mediation and/or elicitation of neurodegenerative diseases during the aging process, a growing body of evidence indicates that ROS may mediate artificial neurotoxicity, such as chemotherapy induced cognitive impairment and peripheral neuropathy. The side effects of anticancer drugs in the nervous system can be too severe to continue chemotherapy, and early termination of therapy may in turn affect the lifespan of the cancer patient. An important and promising area for continued research may thus be in the development of neuroprotective drugs against chemotherapy-induced nervous system side effects.
